# Management approach of patients with violent and aggressive behaviour in a district hospital setting in South Africa

**DOI:** 10.4102/safp.v63i1.5393

**Published:** 2021-10-27

**Authors:** Oladele V. Adeniyi, Ntandazo Puzi

**Affiliations:** 1 Department of Family Medicine, Faculty of Health Sciences, Walter Sisulu University, Mthatha, South Africa; 2 Department of Family Medicine, Faculty of Health Sciences, Cecilia Makiwane Hospital, East London, South Africa; 3 Department of Psychiatry, Faculty of Health Sciences, Walter Sisulu University, Mthatha, South Africa; 4 Department of Psychiatry, Faculty of Health Sciences, Cecilia Makiwane Hospital, East London, South Africa

**Keywords:** aggressive and violent behaviour, assisted user, emergency centres, involuntary user, *Mental Health Care Act*

## Abstract

Aggressive and violent behaviour is very common in the hospital setting. Simple agitation may unpredictably progress to overt aggression and violence by any patient in the emergency centres (ECs). Aggressive behaviour often manifests in forms of verbally abusive language, verbal threats and intimidating physical behaviour. Violent behaviour comprises the intentional use of physical force or power, threatened or actual, against self (suicidal), or another (homicidal) or properties, group or community, that could potentially result in injuries, death, psychological harm or deprivation. Therefore, individuals with unusual agitation and aggression should be treated as an emergency in both the community and healthcare settings in order to mitigate the progression to physical violence. Whilst the incidence and prevalence of aggressive and violent behaviour are higher in individuals with an underlying mental disorder, substance use disorder or comorbid mental disorder and substance use disorder, other individuals can also present with these behaviours in the ECs. Therefore, the front-line clinicians must be knowledgeable and competent in managing patients with aggressive behaviour with a view to de-escalate the situation and preventing or curtailing violence. This paper presents an evidence-based approach for managing patients with aggressive and violent behaviour, including a review of the steps for admitting patients for assisted or involuntary care.

## Introduction

The emergency centres (ECs), otherwise known as casualty, in hospitals serve as the entry point for the majority of individuals with new-onset or persistent violent and aggressive behaviour. Healthcare workers are often victims of violent and aggressive behaviours of their patients. However, there are limited evidence-based intervention strategies to guide the management of patients with aggressive and violent behaviour in acute hospital settings.^1^ Violence and aggression comprise a wide range of behaviours or actions, which can potentially cause harm, hurt or injury by someone to another person.^2^ Violent and aggressive patients have the intention to dominate another person; as such, they express anger, defensive behaviour, verbal abuse, derogatory remarks, threats or non-verbal gestures.^2^

Patients can express a wide range of violent and aggressive behaviours in the ECs. Aggression is described as a disposition towards instilling fear or flight in another person. Aggression includes all acts of hostilities toward becoming violent. Verbal aggression is very common and ranges from angry outbursts, loud shouts and noises, to outright use of verbal threats without real physical harm. The patients’ tone of voice can be a warning sign of imminent violence. According to the World Health Organization (WHO), violence is an intentional use of physical force or power, threatened or actual, against self, or another individual, group or community, that could potentially result in injuries, death, psychological harm or deprivation.^3^ Violence can be directed at individuals or properties. Violence towards objects can occur through slamming of doors, hitting of furniture, setting properties on fire and a host of other things. Violence towards another person can occur through threatening gestures or direct attack on another person causing serious bodily injuries or forcing someone into compromising or undesirable actions such as rape or sodomy.

## Incidence and prevalence of violence and aggression in hospital settings

Aggression and violence from patients are the commonest causes of workplace violence and have reached epidemic proportions worldwide.^4,5,6,7^ According to the National Institute of Health and Care Excellence (NICE), emergency departments and psychiatric units experience far more violence and aggression than any other healthcare settings.^2^ Healthcare workers experience a wide range of violence from patients and/or family members or guardians whilst performing clinical duties.

Verbal assault has been the predominant form of violence reported by the majority of healthcare workers and ranged from 58.0% in Australia to 100.0% in Brazil.^7^ Significantly more female healthcare workers and specifically nurses (82.0%) have experienced more verbal abuse from their patients than their male counterparts. Nurses are three times more likely to experience aggressive and violent patient events whilst performing their duties.^2^ Between 35.0% and 80.0% of healthcare workers have experienced physical assault at least once in their practice. In addition, psychological assaults range from 32.2% in Europe to 67.0% in Australia.^7^ Men are the main perpetrators of physical threats (63.0%) and assaults (52.0%) against the healthcare workers.^5^

Violence and aggression in the hospital setting reflect the broader complex dynamics of violence in the general South African communities. Mahlangu et al.^6^ reported 66.7% of healthcare workers had experienced at least one form of violent and aggressive behaviour in their practice. In general, female healthcare workers experienced violence far more than men, and nurses in particular (66.7%), experienced more violent events than their male counterparts in a South African study.^6^

## Predictors of aggressive and violent behaviour

There is consensus amongst researchers that there is a positive association between the underlying mental disorders (such as bipolar disorder and schizophrenia) and violent behaviour. According to the NICE Expert Committee Report, the life-time prevalence of violence in non-psychiatric population of 7.3% was lower than those with underlying mental illness of 16.1%. However, individuals with substance use disorders were more likely to be violent (35%). The prevalence of violent behaviour increased to 43.6% in individuals with substance use disorder and comorbid mental disorders.^2^ The tendency towards violent behaviour increased in the presence of substance misuse, irrespective of the presence of underlying mental disorders.

Whilst the attending clinicians (doctors and nurses) should take necessary precautions in approaching patients with underlying mental disorders in the ECs, a more cautious approach to individuals with a history of substance misuse, whether with comorbid mental disorders or not, is recommended.^8^ The attending doctor must obtain a comprehensive history, which includes the psychiatric history either from the patient or collateral sources with a view of uncovering the underlying condition(s). Medical history of violent and aggressive behaviour could give insight into future recurrences. The history should explore the pre-morbid state, ongoing medical conditions, personality disorders, mental conditions, substance use and psychological issues of the patient. Table 1^9,10^ provides a comprehensive but non-exhaustive list of conditions that are associated with aggressive and violent behaviour in patients. The attending doctor should not always assume that the aggression is because of the mental illness. As such, a thorough history and examination are recommended for each episode of aggressive and violent behaviour at presentation in the ECs.

**TABLE 1:** Medical conditions associated with aggressive and violent behaviours.

Causes	Associated conditions
Psychiatric	Psychosis, agitated depression, mania, severe anxiety, dementia, previous head injury, intellectual disability, autistic spectrum disorder, dark triad of personality (narcissistic, psychopathy and Machiavellianism), and other developmental disorders.
Psychological factors	High levels of impulsivity and antagonism, excessive reaction to rejection or insult, frustration, poor tolerance, and maladaptive coping skills
Physical	Acute medical illness including various delirium, human immunodeficiency virus-associated neurologic deficit, epilepsy (pre-, intra-, and post-ictal), intracranial lesions, and head injury.
Substance misuse/intoxications	Common substances: alcohol, cannabis, methaqualone (mandrax), stimulants: cocaine, methamphetamine (tik), and methcaninone (cat).
Metabolic abnormalities	Thiamine deficiency, hyponatraemia, hypercalcaemia
Hypoxia, hypercarbia	Pneumonia, deteriorating chronic airway disease
Organ failure	Liver or kidney failure
Withdrawal syndrome	Alcohol (delirium tremens), benzodiazepines
In pregnant women	Labour, obstetric complications, sepsis, organ failure, substances and mental disorders.

*Source*: Adapted from Fulde G, Preisz P. Managing aggressive and violent patients. Australian Prescriber. 2011;34(4):116–118. https://doi.org/10.18773/austprescr.2011.061; South African National Department of Health, South Africa; Essential Drugs Programme. Primary healthcare standard treatment guideline and essential medicine list. 7th ed. Pretoria, South Africa: South African National Department of Health; 2020.

In addition, a mental state examination (Table 2^11^) and the general physical examination should be attempted in the emergency unit before sedating the patient. This often proves difficult to accomplish in the context of a violent and aggressive patient; however, the attending clinician should document his or her attempt at accomplishing this task including the findings. The goal of the clinician is to uncover the underlying aetiology of the person’s aggressive and violent behaviour. The attending clinician should also be aware that the clinical assessment of patients with violent and aggressive behaviour is a dynamic process. As such, periodic evaluation of the patient is recommended.

**TABLE 2:** Mental state examination.

Domain	Comments
Appearance and behaviour	Provide an objective description of the appearance of the patient. Level of alertness, vigilance, attentiveness or distraction, involuntary movements, motor activities, response to interactions and self-care.
Communication	Assess patient’s understanding, expression of words, content of speech and document any impairments.
Mood and emotions	Assess the mood. Is the patient elated? Ask about enjoyment, hopelessness, guilt, self-harm or suicide ideation. Explore emotions such as anxiety, fear or anger, and features of depression such as sleep and appetite.
Perceptions	Assess patient’s overt reactions to hallucinations – visions, voices, tactile or other modalities.
Thoughts	Explore any thought abnormalities in the patient: paranoid, grandiose, or nihilistic. Also, ask if the patient is treated well by others or any other concerns.
Cognition	Explore the patient’s orientation, memory, attention (days of the week or months or year backwards), reasoning and logical thought.
Insight	Does the patient realise that there is a problem?
Risk	Explore the risk of harm to self or others, suicide, absconding, self-neglect, exploitation
Judgement	Does the patient have the capacity to decide about own health or any specific decision?

*Source*: Adapted from Harwood RH. How to deal with violent and aggressive patients in acute medical settings. J Roy Coll Phys Edinb. 2017;47(2):176–182. https://doi.org/10.4997/JRCPE.2017.218.

## Investigations required to exclude a general medical condition

Rational use of laboratory investigations has become critical in the light of the limited budget and increasing health expenditures. However, the evidence-based decision on the initial work-up of patients with aggressive and violent behaviour should target common conditions that are prevalent in the population (Table 1). Investigations should be guided by the attending doctor’s clinical findings (from the history, general physical and mental state examination). Urine dipstick, glucose test, white cell counts and differentials, sodium and creatinine, thyroid-stimulating hormone (TSH), rapid test for human immunodeficiency virus (HIV) and rapid plasma regain (RPR) or venereal disease research laboratory (VDRL) test are recommended. Urine sample for toxic screens for common recreational drugs should be undertaken. A pregnancy test is mandatory for female patients in the reproductive age group. Given the high prevalence of HIV in South Africa, HIV test is mandatory in patients with new-onset behavioural changes, and these patients lack the capacity to consent, so capacity is waivered in the emergency setting.^12^ There may be clinical indications for vitamin B12, B1 and red cell folate assays in selected patients.^2^

However, additional investigations may be necessary based on the clinical findings in the patient. Lumbar puncture (LP) is indicated in patients with clinical suspicion of meningitis, provided there is no contraindication to LP. Patients with a positive serum VDRL or RPR would require a cerebrospinal fluid VDRL test to exclude neurosyphilis. Also, patients with HIV WHO clinical stage 3 and 4 diseases would benefit from LP. A chest X-ray can be done if there is a history of current or previous pulmonary tuberculosis (PTB) or constitutional symptoms or if diagnosed with HIV. Computed tomography (CT) of the brain is indicated if there are altered levels of consciousness, any new focal neurological deficit, new onset seizures, history of alcohol abuse and unexplained disorientation, and clinical suspicion of meningitis with contraindications to LP.^12^

## Management approach for violent and aggressive patients

### Non-pharmacological

In the South African context, the essential drug list of the National Department of Health provides a guide for managing behaviourally disturbed patients. As such, clinicians working in the EC should be well equipped to manage patients with aggressive and violent behaviours. However, few challenges in the South African context range from emergency room architectural design challenges, high patient-to-nurse ratios, lack of security personnel, high prevalence of substance abuse, high crime rates in the general population, and limited medication options.^13^

The primary goal of any intervention towards agitated behaviour is to ensure safety, facilitate assessment of underlying problems and prevent further escalation, through achieving calmness and collaboration.^10,14^ Minimisation of risk to self, others and environment should be the primary aim of all interventions. Common non-pharmacological interventions can be grouped into educational, interpersonal, environmental, and physical responses targeted at the pre-event, event, or post-event phase (Haddon matrix).

The Haddon matrix (Table 3^1^) has been widely used in conceptualising injury prevention threats and modelling solutions.^1^ It consists of three different phases of an injury (pre-event, event and post-event) and the influencing factors (host, agent/vehicle physical environment, social environment factors). Host factors relate to the person or persons at risk of injury (doctors, nurses, hospital personnel and community members). The agent of injury refers to the host (aggressive patient/visitor) through a vehicle (inanimate object) or vector (person or another animal/organism). Physical environment refers to the actual setting (hospital) where the injury occurs. Sociocultural and legal norms of a community constitute the social environment. The use of less restrictive and less intrusive treatment interventions should be encouraged at all times.^10^

**TABLE 3:** Haddon matrix in relation to the management of the aggressive patient.

Physical	Host (staff member/employee)	Agent/vector (aggressive patient/visitor)	Environmental factors	
			Physical	Socioeconomic/social
Pre-event	Education and training Raised awareness Communication and de-escalation Situational awareness Conflict resolution Risk assessments Advanced warning assessments Removal of potential weapons	Policy communication, for example, zero tolerance Communication re-waiting times Provision of clear guidelines and expectations	Physical structures Signage Information availability Security/police/camera visibility Limited visibility of medication areas, valuables Adequate lighting Egress accessibility Metal detectors/weapons’ assessment Use of safety glass, acrylic windows	Organisational policies Community awareness Publicity campaigns Adequate staffing Adequate legislative protection Established procedures for dealing with violent events Management of risk and trigger factors – support limitation of alcohol, address ED overcrowding, poverty initiatives
During event	Initiate appropriate action, for example, activate alarms, remove self from scene Engage in de-escalation, request assistance, self defence Protect self, patients, others	Clearly communicate unacceptability of behaviour Initiate restraint or behavioural protocols Request security/police assistance Isolate perpetrator from others Initiate prosecution	Utilise specialised areas such as quiet rooms, separate waiting areas, low stimulus, seclusion or behavioural units Appropriate resources should be available and accessible Maintain safety of others in the immediate area	Code-based responses/framework Team response/behavioural emergency team/rapid response team, etc. Recognised protocol Workplace culture of non-acceptance and expected response to all incidents
Post-event	Reporting systems – incident report Feedback and follow-up Medical and counselling availability Peer support	Potential responses Barring or trespassing individuals Follow-up solicitor or manager letters Prosecution Investigation of underlying factors Initiation of alerts or warnings	Identification of any physical contributions, e.g., lack of space, overcrowding, inability to safely exit, accessibility to weapons of convenience	Recognition of trigger factors and evaluation of process issue Review of response processes and efficacy

*Source*: Richardson SK, Ardagh MW, Morrison R, Grainger PC. Management of the aggressive emergency department patient: Non-pharmacological perspectives and evidence base. OAEM. 2019;2019(11):271–290. https://doi.org/10.2147/OAEM.S192884

First, the attending doctor should prepare, anticipate and readily prevent aggression in the EC. Certain individuals (those with a history of substance misuse, previous violence and state patients on leave of absence) are at higher risk of becoming aggressive and violent. The EC staff should be aware of the early signs of aggression from these patients or others. Every health facility must have laid down protocols to ensure safety of all patients and staff. The protocol should contain the triage plan for early signs of aggression and the roles of each staff in such a situation. There must also be back-up plans for the safety of staff, patients and properties, such as security personnel, South African police services and the emergency medical personnel. Each health facility must have a designated area/room for calming down aggressive and violent patients and regular monitoring.^10^

The next step is to de-escalate and contain the patient. The attending doctor must be very calm, confident, reassuring and keep an open disposition. The following recommendations will help to keep the attending doctor safe^10^:

- Do not turn his or her back on the patient.
- Avoid direct eye contact with the patient.
- Do not reason with the patient.
- Do not challenge patient’s delusions or touch the patient.
- Set clear limits regarding the behaviour.

Manual restraint may be necessary to administer treatment to the patient. Mechanical restraints should be used only when absolutely necessary to protect the patient and others in an acute setting for as short a period as possible.^10^ Types, sites and duration of any restraints used must be documented with 15-min monitoring of vital signs, the mental state, restraint sites, and reasons for use.^2,10^

The *Mental Health Care Act* (MHCA) Form 48 should be completed by the attending clinician and submission be made to the Mental Health Review Board to approve the use of mechanical restraint in the patient.^15^ Non-pharmacological interventions should preferably precede pharmacological interventions in patients with aggressive and violent behaviours. The attending doctor should be aware of high-risk patients: those with a history of violence, substance misuse, and state patients on leave of absence. Every hospital must have a designated calming area (suitable for monitoring) for attending to high-risk patients including those with features of aggression. The attending doctor must secure the help of other staff including hospital security, South African Police Service and emergency medical service, and assign clear responsibilities.

Involuntary and assisted admission of a mental health care user (patient) (MHCU) for treatment, care, and rehabilitation is both a medical and a legal process.^12^ Any admission should be done according to the *Mental Health Care Act,* 17 of 2002.^15^ The indications for assisted or involuntary admission are^12^ as follows:

- There must be a presence of a mental illness.
- There must be a high likelihood to cause serious harm to self or others (suicidal/homicidal) or to cause harm to their financial interests or reputation.
- The person cannot make an informed decision on the need for treatment and rehabilitation.
- The person is not unwilling to receive treatment (does not object – passive consent) in case of the assisted user or the person outrightly objects to receive treatment and rehabilitation in case of involuntary user.

### Guideline for the admission of involuntary and assisted persons under the *Mental Health Care Act,* 2002 (Act No. 17 of 2002)^15^

Table 4^15^ details the various *Mental Health Care Act* forms and their indications. Outlined below are the steps to be followed by the attending clinicians in admitting patients for involuntary or assisted care.

**TABLE 4:** Mental Health Care Act forms for assisted and involuntary admissions.

MHCA form	Responsible individual(s)	Duties
Form 4	Spouse, next of kin, partner, associate, parent or guardian. May be made by a healthcare provider.	Application for admission Applicant must have seen the user in the last 7 days
Form 5 (two forms)	Two mental healthcare practitioners (MHCPs) (one qualified to conduct physical examinations).	Record of findings by two MHCPs (must not be the person making the application). At least one dated the same date as Form 4. Other within 24 h of Form 4
Form 7	HHE	Notice by HHE copies to applicant, user and Review Board
Form 6 (two forms)	Two MHCPs	72-h assessment and findings after HHE has granted permission to admit
Form 8	HHE	Notice by HHE to review board requesting approval for further involuntary care, treatment and rehabilitation on an in-patient basis
Form 9	HHE	Notice by HHE to review board requesting approval for further involuntary care, treatment and rehabilitation on an outpatient basis
Form 11	HHE	Transfer of assisted or involuntary MHCU on an in-patient basis to another health establishment
Form 1	Healthcare practitioner	Report to MHRB on provision of care, treatment and rehabilitation without consent or emergency admission.
Form 22	SAPS	Handing over custody by the South African Police Services (SAPS) of a person suspected of being mentally ill and likely to inflict serious harm to himself or herself or others

*Source*: Mental Health Care Act 17 of 2002. [cited 2021 Aug 12]. Available from: https://www.hpcsa.co.za/Uploads/Legal/legislation/mental_health_care_act_17_of_2002.pdf

MHCU, mental health care user; MHRB, Mental Health Review Board; MHCA, *Mental Health Care Act*; HHE, Head of Health Establishment.

STEP 1: Family/guardian/associate to apply for admission on *Mental Health Care Act* Form 04. However, the mental healthcare practitioner (MHCP), in the absence of family/guardian/associate, can apply for admission on *Mental Health Care Act* Form 04 after documenting steps to get one of the listed persons. The MHCP must not be the attending doctor.

STEP 2: Person to be assessed by two MHCPs. Examinations and findings should be recorded on *Mental Health Care Act* Form 05 (X2). One of the practitioners should be able to conduct a physical examination. The second MHCPs can be a nurse or another doctor.

STEP 3: MHCP must submit *Mental Health Care Act* Forms 04 and 05 (X2) to the Head of Health Establishment (HHE) for approval for admission.

STEP 4: The HHE should decide on whether or not to provide further care and to give notice of consent to such care on *Mental Health Care Act* Form 07.

STEP 5: Person can now be admitted or treated for 72 h without his or her consent.

STEP 6: Person should be assessed every 24 h for 72 h.

STEP 7: Two MHCPs will re-assess the person after 72 h have elapsed, and the examinations and findings should be recorded on *Mental Health Care Act* Form 06 (X 2).

STEP 8: MHCP should submit *Mental Health Care Act* Form 06 to the HHE.

STEP 9: The HHE decides whether the person needs to be further treated as an outpatient (*Mental Health Care Act* Form 09), in-patient (*Mental Health Care Act* Form 08), or to be discharged (*Men tal Health Care Act* Form 03), and gives notice to the Mental Health Review Board on *Mental Health Care Act* Form (depicted in the parenthesis above).

STEP 10: If further treatment is required as an in-patient, the person must be transferred to a designated mental healthcare facility. The HHE should complete *Mental Health Care Act* Form 11.

### Pharmacological management

Pharmacological management can be conceptualised in the acute setting (immediate sedation) and for the long-term prevention in persistently violent and aggressive patients.

### Acute setting

The aim is to reach calmness within a maximum period of 2 h whilst avoiding adverse effects. Olanzapine was the most frequently studied drug in a systematic review by Bak et al.^14^ Changes at 2 h showed the strongest effect for haloperidol plus promethazine, risperidone, olanzapine, droperidol and aripiprazole. Adverse effects are most prominent for haloperidol and haloperidol plus lorazepam.^16^ Oral Benzodiazepines; Lorazepam, oral, 0.5 mg – 2.0 mg, or Clonazepam, oral 0.5 mg – 2.0 mg, or Diazepam, oral, 5 mg – 10 mg or Midazolam, buccal, 7.5 mg – 15.0 mg, should be prioritised first, according to the Essential Drug List.^10^ Oral administration of treatment is the safest route.

For patients who did not respond to a repeated oral sedation, refuse oral sedation, or place themselves and others at significant risk, intramuscular sedation for rapid tranquilisation is recommended. Rapid tranquilisation with a short-acting benzodiazepines, for example: Lorazepam 0.5 mg – 2.0 mg immediately or Midazolam 7.5 mg – 15.0 mg immediately or Clonazepam 0.5 mg – 2.0 mg immediately. Repeat after 30–60 min if needed or Haloperidol, IM, 5.0 mg, immediately and promethazine, IM 25.0 mg – 50.0 mg (in the elderly 25.0 mg). Patients with respiratory insufficiency should be given haloperidol instead of benzodiazepines. Patients with underlying psychosis can be given haloperidol and promethazine as first-line treatment rather than benzodiazepines. In patients suspected of alcohol intoxication, thiamine, oral 300 mg, should be added and continued daily for 14 days.^10^

Always monitor the vital signs of a sedated patient. Rapid tranquillisation may cause cardiovascular collapse, respiratory depression, neuroleptic malignant syndrome and acute dystonic reactions. Sedation of children with psychotropic agents should only be considered in extreme cases and only after consultation with a psychiatrist. The current trend is for use of newer, yet equally potent agents, with better side effect profiles over traditional agents like haloperidol, clotiapine (etomine) and zuclopenthixol acetate injection (clopixol acuphase); yet most of the newer agents are not all available in South Africa, especially in the public sector.

**FIGURE 1:**
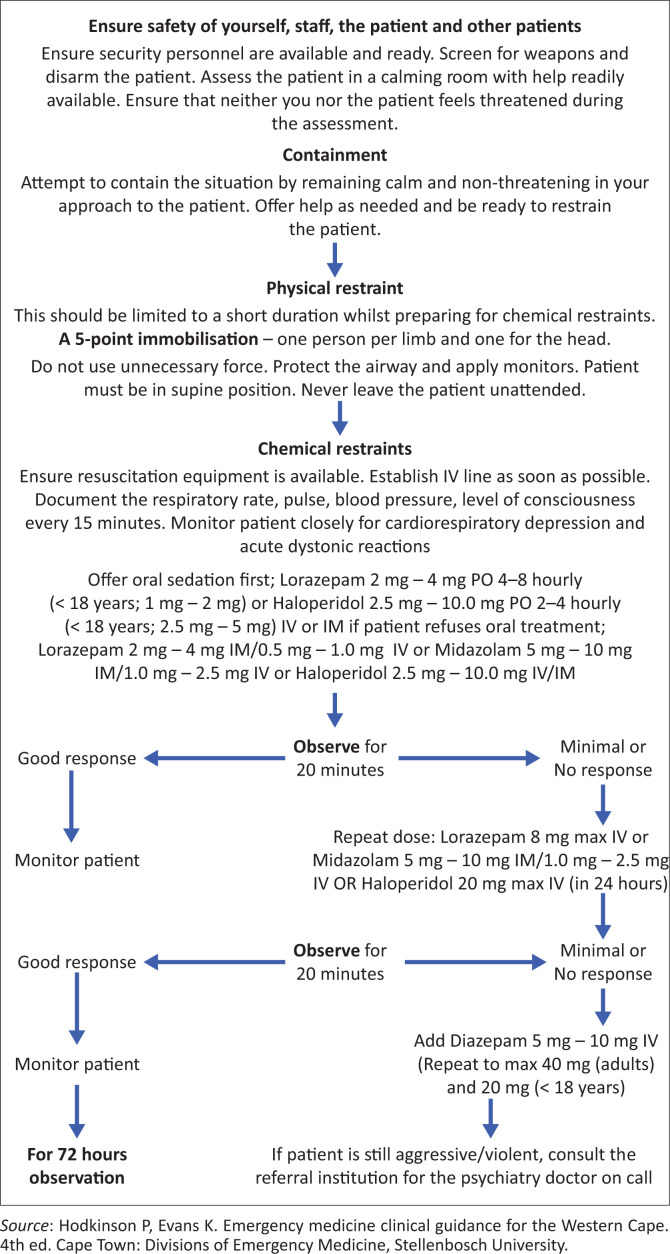
Algorithm for managing aggressive and violent patient in a district hospital.

Haloperidol intramuscular injection often combined with Lorazepam 2 mg – 4 mg is still the mainstay of care in the ECs in South Africa. Oversedation, dystonic reactions (laryngospasm, oculogyric crisis and torticollis) and akathisia (inner feeling of restlessness) are common unwanted effects with haloperidol. Very little published data supports the use of zuclopenthixol acetate injection (clopixol acuphase), and it should not be used as a first line for rapid tranquilisation. Avoid the use of zuclopenthixol acetate on anti-psychotic naïve patients, patients with known cardiac conditions and in patients with a history of extrapyramidal side effects. Its onset of action is often delayed and its effects may last for 2–3 days. Therefore, zuclopenthixol acetate (clopixol acuphase) should only be used after an acutely psychotic patient has required repeated injections of short-acting antipsychotics such as haloperidol or olanzapine and/or sedative drugs such as lorazepam, and these have not been effective.^17^ Dose ranges between 50 mg and 150 mg, repeated, if necessary, after 2 days or 3 days. Figure 1^12^ summarises the approach to the management of an aggressive and violent patient in the EC, which can be easily implemented at all the district hospitals in the country.

In a review of the literature by Carpenter et al.,^16^ there was no significant evidence to support the use of clotiapine (etomine) rather than other ‘standard’ or ‘non-standard’ treatments for the management of acute psychotic illness. This further points to the fact that good randomised controlled trials (RCTs) are needed.

## Persistent violence and aggression in chronic psychiatric patients

Persistent violence and aggression are very common amongst chronic psychiatric patients and state patients, substance abusers, major neurocognitive disorders and patients with intellectual disability.^18^ These patients are mostly encountered in psychiatric inpatients and forensic psychiatry settings. The move towards de-institutionalisation, with its advantages and disadvantages, has resulted in individuals with serious mental illness living in the community and having frequent visits to local ECs. State patients (a person so classified by a court directive in terms of section 77(6)(a)(i) or 78(6)(i)(aa) of the *Criminal Procedure Act* and detained in a psychiatric hospital or a prison pending the decision of a judge in chambers in terms of section 47 of the MHCA (2002) are often given leave of absence, conditional or unconditional discharges.^15^ During these periods, they are often required to access treatment, care and rehabilitation at their local hospitals. Studies have also shown that when these individuals are treated, the incidence of violent behaviour decreases significantly.

Three categories could be used to define these aggressive acts: psychotic, impulsive, and predatory (also called organised or instrumental) (Table 5^18^). Impulsive violence is the most common form seen amongst chronic psychiatric inpatients despite the high prevalence of psychosis.

**TABLE 5:** Proportion of aggressive acts.

Sub-types	Characteristics	Percentage
Psychotic	Behaviour is motivated by positive symptoms of psychosis (hallucinations, delusions)	17
Predatory/Organised	Planned behaviour with clear goals in mind, for example; intimidation, retribution, monetary or material gain. Behaviour not obviously a response to threat or provocation. Often accompanied by limited autonomic arousal.	29
Impulsive	Behaviour is precipitated by provocation, threat and stress. Often associated with fear, anger and frustration. High levels of autonomic arousal.	54

*Source*: Adapted from Stahl SM. Deconstructing violence as a medical syndrome: Mapping psychotic, impulsive, and predatory subtypes to malfunctioning brain circuits. CNS Spectrums. 2014;19(5):357–365. https://doi.org/10.1017/S1092852914000522.

Evidence is most robust for psychotic and impulsive aggression. Organised or instrumental violence is generally not amenable to pharmacotherapy and requires behavioural techniques and custodial management. Psychotic violence and aggression are the direct products of poorly controlled positive symptoms of psychosis; therefore, their treatment is consistent with known algorithms for managing inadequate responders.^2^

Clozapine should be considered after non-response to at least two adequate trials of antipsychotics and clinicians should familiarise themselves with initiation of clozapine therapy, management of patients on clozapine and recognition of clozapine side effects. Patients with a diagnosis of schizoaffective disorder, bipolar type, may not respond sufficiently to anti-psychotic monotherapy, and mood stabilisation is often necessary to control partially remitted mania or hypomania that continues to drive psychotic symptoms.^19^ Clozapine also emerges as the preferred agent for impulsive violence and aggression, and its anti-aggressive property in these individuals is independent of its impact on psychotic symptoms. Adjunctive options include sodium valproate, centrally acting beta-adrenergic antagonists, lithium and selective serotonin re-uptake inhibitors (SSRIs) antidepressants. Data for lithium in schizophrenia patients are limited.^20^

Based on the small number of RCTs, only the centrally acting beta-blockers (Propranolol) had strong evidence for efficacy for non-psychotic violent and aggressive patients with traumatic brain injury.^20^ Carbamazepine and sodium valproate seem effective for agitation and aggression in traumatic brain injury and are recommended as first-line treatment with sodium valproate having a lesser side effect profile. Amongst the pharmacologic options for persistent aggression in patients with major neurocognitive disorder, the strongest evidence points to the benefits of acetylcholine esterase inhibitors (AChEls) for neuropsychiatric symptoms of mild-to-moderate Alzheimer’s disease. Mematine (an *N*-methyl-*D*-aspartate [NMDA]) receptor antagonist used in the management of Alzheimer’s disease) has shown efficacy for both aggression and loss of appetite in patients with Alzheimer’s disease. It is effective both as monotherapy and when combined with AChEIs. Selective serotonin re-uptake inhibitors have also shown some efficacy. Anti-psychotics are associated with the risk of mortality and morbidity in these patients and should be used with caution. First-generation antipsychotics should be avoided at all costs, if possible.

In conclusion, clinicians working at the district hospitals must be aware that patients with aggressive and violent behaviour often present at the ECs. They must receive training in de-escalation protocol and the management approach for patients with aggressive and violent behaviour. Oral treatment should be prioritised when feasible. However, mechanical restraints can be applied minimally with a view of achieving sedation in some patients through parenteral route.
